# Review on Atrial Fibrillation

**DOI:** 10.19102/icrm.2017.081209

**Published:** 2017-12-15

**Authors:** Rahul N. Doshi

**Affiliations:** ^1^University of Southern California Keck School of Medicine, Los Angeles, CA, USA

**Keywords:** Atrial fibrillation, review

## Introduction

The year 2017 has been filled with news, controversy, and turmoil on many stages, in the realms of politics, entertainment, and economics—both around the world and locally. While original investigation regarding atrial fibrillation (AF) may not be one of these areas, nevertheless, this year was still filled with very significant contributions to our field.

AF is still the largest area of investigation in the field of clinical cardiac electrophysiology. At the time of this writing, if one enters the term “atrial fibrillation” into the search field of PubMed and limits their search to publications in the year 2017, there are 4,867 results. This is compared with only 1,394 entries that appear when using the term “sudden cardiac death” and the same search criteria. Even if one limits their interest to data from clinical trials, there is a mountain of literature published annually. Thus, the following should be viewed as only a limited representation rather than a comprehensive review of the literature published surrounding AF.

## Atrial fibrillation: pandemic proportions

It has long been recognized that AF is an arrhythmia of pandemic proportions, with the lifetime risk of developing the disease after the age of 40 hovering at around 25%.^1^ The number of individuals with AF in the United States is expected to double by the year 2050.^2^ “AF begets AF” is a long-heralded mantra reflective of the idea that AF is a progressive diease.^3^ Padfield et al., in a manuscript published in June of this year, sought to determine the rate of progression from paroxysmal to persistent AF over a 10-year period.^4^ Analyzing data from the Canadian Registry of Atrial Fibrillation, they demonstrated that more than 50% of patients with paroxysmal AF over 10 years will either have progressed to having persistent AF, or have died. The rate of progression was 36.3% at 10 years. This sobering result suggests that we have a long way to effectively halt the progression of this disease despite early recognition.

AF also remains intertwined with other disease epidemics that continue to plague our health care system. The association between AF and congestive heart failure remains an incredibly common finding, with roughly 40% of patients having one of these conditions being likely to develop the other.^5^ Cherian and colleagues examined the effects of AF on patients with heart failure within the ORBIT-AF registry.^6^ They report that, among patients with AF, those with heart failure had a similar rate of stroke but a higher risk of death or hospitalization [hazard ratio (HR): 1.69 and 1.31, respectively) as compared with those without heart failure. In patients with a left ventricular ejection fraction < 40%, the HRs were 2.06 and 1.38, respectively. This was despite a good background of heart failure therapy. These results, while equally concerning, are notably both the result of detrimental hemodynamic effects, which may support the notion that the treatment of AF in this population should include a rhythm control strategy versus methods aimed solely at preventing thromboembolism.

Another growing health problem is the obesity epidemic. Interestingly, there are reports that detail a protective effect of obesity regarding AF, termed the “obesity paradox.”^7^ Balla and collaborators examined the relationship of body mass index (BMI) and the risk of stroke in patients with AF^8^ in patients treated in the ROCKET AF trial.^9^ They found that individuals with a BMI ≥ 35 kg/m^2^ had a lower risk of stroke when compared with their normal-weight counterparts, whether on rivaroxaban or warfarin (HR: 0.62 and 0.48, respectively). While the mechanism of this effect is secondary to increased time in the therapeutic range in the warfarin group or is related with favorable effects on the potential prothrombotic state, it remains a fact that despite this obesity paradox, there is research linking obesity with an increased risk of the development of AF^10^ and the progression of paroxysmal AF to persistent AF.^11^

## Atrial fibrillation and stroke prevention

The last several years has seen an increase in a focus on the prevention of thromboembolism secondary to AF corresponding with the growing number of treatment options beyond warfarin. This year was no exception. Boersma et al., on behalf of the EWOLUTION investigators, reported on their one-year follow-up data of left atrial appendage (LAA) occlusion with the WATCHMAN^™^ device (Boston Scientific, Natick, MA, USA).^12^ This trial included more than 1,000 patients who met the criteria for LAA occlusion according to the European Society of Cardiology guidelines^13^ with or without a contraindication for anticoagulation. In the trial, 73% of patients were deemed unsuitable for anticoagulation. Thus, a mix of patients taking warfarin (16%), novel oral anticoagulants (11%), dual antiplatelet therapy (60%), single antiplatelet therapy (7%), and no therapy (6%) in the immediate peri-procedure period were included in this study. Subsequent findings indicated a high level of procedural success (98.5%) and low residual leaks > 5mm (99.5%), with device thrombus present in 3.7% of participants. Notably, the presence of device thrombus did not correlate with anticoagulant regimen. Furthermore, the annual stroke rate was 1.1% despite a high baseline CHA_2_DS_2_-VASc score of 4.5. Thus, LAA occlusion with the WATCHMAN^™^ device (Boston Scientific, Natick, MA, USA) was deemed effective at preventing stroke even without peri-procedural anticoagulation. These data also pave the way for the ASAP-TOO trial, a randomized prospective trial investigating the effectiveness of LAA occlusion with the same device in patients who are labeled unsuitable for oral anticoagulation.^14^

Hot off the press are the five-year outcomes data from the PROTECT and PREVAIL trials, which examined again the WATCHMAN^™^ device (Boston Scientific, Natick, MA, USA) versus long-term warfarin therapy in patients with nonvalvular AF.^15^ Reddy and colleagues report on findings from more than 1,100 patients who were followed for five years, and demonstrate noninferiority in the composite endpoint of stroke, systemic embolism, or cardiovascular/unexplained death when compared with warfarin. However, much like the previous trials, there were significant decreases in hemorrhagic stroke, disabling stroke, cardiovascular/unexplained death, total mortality, and post-procedure bleeding. The most significant decrease was in all-cause mortality, with a demonstrated HR of 0.73 for the device as compared with warfarin. These results are extremely encouraging and, although we still do not yet have a head-to-head comparison between the WATCHMAN^™^ device (Boston Scientific, Natick, MA, USA) and novel oral anticoagulation therapy, it appears that LAA occlusion therapy should be included in our arsenal for the prevention of stroke in patients with nonvalvular AF.

## Predicting atrial fibrillation

While our therapy choices for stroke prevention continue to grow, many patients will only present for the first time with a significant thromboembolic event.^16^ This underscores the importance of identifying patients at risk for developing AF in a predictive manner. The PREDATE AF study presented by Nasir et al. prospectively evaluated the use of the CHA_2_DS_2_-VASc score to predict new-onset AF using implantable loop recorders (REVEAL XT^™^ or Reveal LINQ^™^; Medtronic, Minneapolis, MN, USA) and determined whether this led to the initiation of anticoagulant therapy.^17^ In almost 250 patients with a CHA_2_DS_2_-VASc score ≥ 2, 22.4% were noted to have new F, defined as an episode lasting longer than six minutes, over the 18-month study period. Of these individuals, 76.4% were subsequently started on oral anticoagulation. There was no demonstrated increase in incidence of AF correlated with an increase in CHA_2_DS_2_-VASc score, but the study was likely underpowered to detect such a finding. These data, taken together with those of trials presented previously, such as the ASSERT-II trial,^18^ support the use of implantable monitors to detect AF in high-risk patients.

An exciting development in the ability to predict AF is utilizing our knowledge of mechanisms or arrhythmia onset or initiation. It has been demonstrated that the autonomic nervous system plays a vital role in the modulation of cardiac arrhythmias, from sudden cardiac death to AF.^19^ Increased stellate ganglia sympathetic nerve activity has been demonstrated to be associated with atrial arrhythmias^20^ but requires invasive measurements to elucidate. Skin sympathetic nerve activity is a potential marker of stellate ganglia activity. Interestingly, a group of researchers from Indiana University has elegantly demonstrated simultaneous measurements of skin sympathetic nerve activity and electrocardiogram recordings.^21^ From this group, Uradu and colleagues further reported based on findings from a small group of patients that skin sympathetic nerve activity increases prior to the onset and termination of atrial tachycardia and AF.^22^ This technology, though only in its infancy, has a significant potential to both predict AF noninvasively and further our understanding of atrial arrhythmias. These findings may lead to the development of treatment algorithms that help to modulate autonomic activity to ensure better control of arrhythmias.

## Atrial fibrillation ablation

The past year has had no shortage of literature regarding the use of catheter ablation as a treatment for AF. While we all eagerly await the next “big thing” with regards to this area, 2017 still provided us with some important data to refine the technology and techniques for AF ablation.

The last several years have solidified pulmonary vein isolation (PVI) as the cornerstone of AF ablation, while suggesting that additional ablation procedures including lines or substrate targeting do not confer an additional benefit. Trials such as the STAR-AF study have suggested that this is true even in patients with persistent AF.^23^ Adding to this literature, Fink et al. published the results of the Alster-LOST-AF trial.^24^ One hundred twenty-four patients with persistent or longstanding persistent AF were randomized in a 1:1 fashion to undergo PVI alone or PVI with substrate modification. They reported a one-year tachyarrhythmia-free incidence of 54% in the PVI-alone group versus 57% in the group who underwent PVI with substrate ablation. Notably, the substrate ablation followed the stepwise approach.^25^ As such, while these results suggest that targeted substrate ablation may not add value to PVI, the low single-procedure success rates underscore the need for further investigation regarding what is the ideal approach for catheter ablation of AF.

Despite success rates that leave room for much improvement, catheter ablation for AF still may be the best treatment for patients with advanced disease, especially those with an increased risk for poor outcomes, such as those with heart failure or cardiomyopathy. Previous trials such as the AATAC trial have demonstrated the superiority of catheter ablation over pharmacologic therapy and associated improvements in left ventricular ejection fraction.^26^ In the CAMERA-MRI trial, Prabhu and colleagues report their results of catheter ablation versus rate control for AF in a population of patients with idiopathic cardiomyopathy.^27^ Specifically, they demonstrate very significant improvements in ejection fraction in the ablation group as compared with in the rate control group (ie, absolute increase of 18% versus 4.4%). Moreover, they indicate that the degree of improvement correlates with a lack of late gadolinium enhancement on cardiac magnetic resonance imaging. The single-procedure success rate in the trial was 56% off antiarrhythmics and 75% on medications. This important finding supports the idea that AF itself is an important cause of left ventricular dysfunction even when there is adequate rate control, and the lack of significant fibrosis predicts recovery. Catheter ablation for AF in this population would expect to lead to significant improvements in overall mortality given the improvements in systolic function.

In regards to ablation protocol, there were multiple reports in 2017 refining current techniques. Aryana et al. report using a time-to-isolation parameter as a means of dosing cryoablation for AF.^28^ They suggest that this approach has similar results to the conventional approach while decreasing the total amount of cryoablation time, as a time-to-isolation on first application of < 60 seconds only requires a single freeze. Also, Cardosos et al. published an important meta-analysis of ablation with uninterrupted novel oral anticoagulant use^29^ demonstrating equivalency to uninterrupted warfarin but with a significantly lower risk of major bleeding.

Finally, the one interesting development in catheter technology in 2017 was the first reported human studies involving a unique diamond-tip catheter with six thermocouples that allows for irrigated ablation with temperature control. Iwasawa and colleagues reported that the use of this catheter is feasible in a single-center series.^30^ The authors suggest that using temperature control allows for an appropriate modulation of power so as to achieve transmurality without the risk of steam pops or char formation. Temperature feedback, of course, is used in traditional nonirrigated ablation and has long been understood to have the greatest correlation with lesion depth in standard radiofrequency (RF) ablation.^31^ Temperature control capabilities have also been used previously in an internally cooled ablation catheter during AF ablation.^32^ The authors of this current study demonstrate a 70% decreased RF time in comparison with that seen with conventional contact force ablation, but a 100% rate of acute procedural success. This catheter design may prove to greatly increase the efficiency of point-by-point catheter ablation.

## 2017 Guidelines for atrial fibrillation

Last but certainly not least, 2017 gave us an updated consensus document for the treatment of AF. The 2017 Heart Rhythm Society/European Heart Rhythm Assocation/European Cardiac Arrhythmia Society/Asia Pacific Heart Rhythm Society/ Sociedad Latinoamericana de Estimulación Cardíaca y Electrofisiología expert consensus statement on catheter and surgical ablation of AF, chaired by Dr. Hugh Calkins, gives us several important new takeaways.^33^ First, the guidelines make a distinction in the treatment of paroxysmal versus persistent AF, that while patients with paroxysmal AF should have symptoms and fail a medication in order to reach a point of consideration for ablation, that it is reasonable to consider ablation as a first-line therapy in patients with persistent AF whether or not they have been tried on an antiarrhythmic drug. The guidelines also clearly state that ablation is reasonable in patients with heart failure or hypertrophic cardiomyopathy. These statements reflect the importance of recognizing that these individuals are at a higher risk of morbidity and mortality associated with AF. Third, the guidelines note clearly that there is strong evidence that ablation improves symptoms and left ventricular function. Lastly, they indicate that the decision to discontinue anticoagulant therapy should be based on the individual risk of thromboembolism and not the clinical outcome of the procedure.

## Conclusion

I hope that this review is useful in summarizing some of the key literature published in 2017. Again, this is simply representative and not all-inclusive of the available topics, and clearly reflects my own biases. I anxiously look forward to new discoveries and innovations for the treatment of AF sure to come in 2018.

## References

[1] Lloyd-Jones DM, Wang TJ, Leip EP (2004). Lifetime risk for development of atrial fibrillation: the Framingham Heart Study. Circulation.

[2] Go AS, Mozaffarian D, Roger VL (2013). Heart disease and stroke statistics–2013 update: a report from the American Heart Association. Circulation.

[3] Wijffels MC, Kirchhof CJ, Dorland R, Allessie MA (1995). Atrial fibrillation begets atrial fibrillation. A study in awake chronically instrumented goats. Circulation.

[4] Padfield GJ, Steinberg C, Swampillai J (2017). Progression of paroxysmal to persistent atrial fibrillation: 10-year follow-up in the Canadian Registry of Atrial Fibrillation. Heart Rhythm.

[5] Wang TJ, Larson MG, Levy D (2003). Temporal relations of atrial fibrillation and congestive heart failure and their joint influence on mortality: the Framingham Heart Study. Circulation.

[6] Cherian TS, Shrader P, Fonarow CG (2017). Effect of atrial fibrillation on mortality, stroke risk, and quality-of-life scores in patients with heart failure (from the Outcomes Registry for Better Informed Treatment of Atrial Fibrillation [ORBIT-AF]). Am J Cardiol.

[7] Overvad TF, Rasmussen LH, Skjøth F, Overvad K, Lip GY, Larsen TB (2013). Body mass index and adverse events in patients with incident atrial fibrillation. Am J Med.

[8] Balla SR, Cyr DD, Lokhnygina Y (2017). Relation of risk of stroke in patients with atrial fibrillation to body mass index (from Patients Treated with Rivaroxaban and Warfarin in the Rivaroxaban Once Daily Oral Direct Factor Xa Inhibition Compared with Vitamin K Antagonism for Prevention of Stroke and Embolism Trial in Atrial Fibrillation Trial). Am J Cardiol.

[9] Patel MR, Mahaffey KW, Garg J (2011). Rivaroxaban versus warfarin in nonvalvular atrial fibrillation. N Engl J Med.

[10] Wanahita N, Messerli FH, Bangalore S, Gami AS, Somers VK, Steinberg JS (2008). Atrial fibrillation and obesity: results of a meta-analysis. Am Heart J.

[11] Tsang TS, Barnes ME, Miyasaka Y (2008). Obesity as a risk factor for the progression of paroxysmal to permanent atrial fibrillation: a longitudinal cohort study of 21 years. Eur Heart J.

[12] Boersma LV, Ince H, Kische S (2017). Efficacy and safety of left atrial appendage closure with WATCHMAN in patients with or without contraindication to oral anticoagulation: 1-year follow-up outcome data of the EWOLUTION trial. Heart Rhythm.

[13] Kirchhof P, Benussi S, Kotecha D (2016). 2016 ESC Guidelines for the management of atrial fibrillation developed in collaboration with EACTS. Eur Heart J.

[14] Holmes DR (2017). The Assessment of the Watchman Device in Patients Unsuitable for Oral Anticoagulation (ASAP-TOO) trial. Am Heart J.

[15] Reddy VY, Doshi SK, Gibson DN (2017). 5-year outcomes after left atrial appendage closure from the PREVAIL and PROTECT AF trials. J Am Coll Cardiol.

[16] Mozaffarian D, Benjamin EJ, Go AS (2015). Heart disease and stroke statistics–2015 update: a report from the American Heart Association. Circulation.

[17] Nasir JM (2017). Predicting Determinants of Atrial Fibrillation or Flutter for Therapy Elucidation in Patients at Risk for Thromboembolic Events (PREDATE AF) Study. Heart Rhythm.

[18] Healey JS, Alings M, Leong-Sit P (2016). Prevalence of sub-clinical atrial fibrillation using an implantable cardiac monitor in patients with cardiovascular risk factors: ASSERT II. Circulation.

[19] Shen MJ, Zipes DP (2014). Role of the autonomic nervous system in modulating cardiac arrhythmias. Circ Res.

[20] Choi EK, Shen MJ, Han S (2010). Intrinsic cardiac nerve activity and paroxysmal atrial tachyarrhythmia in ambulatory dogs. Circulation.

[21] Doytchinova A, Hassel JL, Yuan Y (2017). Simultaneous non-invasive recording of skin sympathetic nerve activity and electrocardiogram. Heart Rhythm.

[22] Uradu A, Wan J, Doytchinova A (2017). Skin sympathetic nerve activity precedes the onset and termination of paroxysmal atrial tachycardia and fibrillation. Heart Rhythm.

[23] Verma A, Mantovan R, Macle L (2010). Substrate and Trigger Ablation for Reduction of Atrial Fibrillation (STAR AF): a randomized, multicentre, international trial. Eur Heart J.

[24] Fink T, Schlüter M, Heeger CH (2017). Stand-alone pulmonary vein isolation versus pulmonary vein isolation with additional substrate modification as index ablation procedures in patients with persistent and long-standing persistent atrial fibrillation: the randomized Alster-LOST-AF Trial (Ablation at St. Georg Hospital for Long-Standing Persistent Atrial Fibrillation). Circ Arrhythm Electrophysiol.

[25] Haïssaguerre M, Hocini M, Sanders P (2005). Catheter ablation of long-lasting persistent atrial fibrillation: clinical outcome and mechanisms of subsequent arrhythmias. J Cardiovasc Electrophysiol.

[26] Di Biase L, Mohanty P, Mohanty S (2016). Ablation versus amiodarone for treatment of persistent atrial fibrillation in patients with congestive heart failure and an implanted device: results from the AATAC multicenter randomized trial. Circulation.

[27] Prabhu S, Taylor AJ, Costello BT (2017). Catheter Ablation Versus Medical Rate Control in Atrial Fibrillation and Systolic Dysfunction. The CAMERA-MRI Study. J Am Coll Cardiol.

[28] Aryana A, Kenigsberg DN, Kowalski M (2017). Verification of a novel atrial fibrillation cryoablation dosing algorithm guided by time-to-pulmonary vein isolation: Results from the Cryo-DOSING Study (Cryoballoon-ablation DOSING Based on the Assessment of Time-to-Effect and Pulmonary Vein Isolation Guidance). Heart Rhythm.

[29] Cardoso R (2017). An updated meta-analysis of novel oral anticoagulants versus vitamin K antagonists for uninterrupted anticoagulation in atrial fibrillation catheter ablation. Heart Rhythm.

[30] Iwasawa J, Koruth JS, Petru J (2017). Temperature-controlled radiofrequency ablation for pulmonary vein isolation in patients with atrial fibrillation. J Am Coll Cardiol.

[31] Nath S, DiMarco JP, Gallop RJ, McRury ID, Haines DE (1996). Effects of dispersive electrode position and surface area on electrical parameters and temperature during radiofrequency catheterablation. Am J Cardiol.

[32] Doshi RN, Oley L, Doshi A, Ceballos S, Mendez F (2012). Single center experience with a closed-loop irrigated ablation catheter for the treatment of human paroxysmal and persistent atrial fibrillation. J Innov Cardiac Rhythm Manage.

[33] Calkins H, Kuck KH, Cappato R (2012). 2012 HRS/EHRA/ECAS expert consensus statement on catheter and surgical ablation of atrial fibrillation: recommendations for patient selection, procedural techniques, patient management and follow-up, definitions, endpoints, and research trial design: a report of the Heart Rhythm Society (HRS) Task Force on Catheter and Surgical Ablation of Atrial Fibrillation. Developed in partnership with the European Heart Rhythm Association (EHRA), a registered branch of the European Society of Cardiology (ESC) and the European Cardiac Arrhythmia Society (ECAS); and in collaboration with the American College of Cardiology (ACC), American Heart Association (AHA), the Asia Pacific Heart Rhythm Society (APHRS), and the Society of Thoracic Surgeons (STS). Endorsed by the governing bodies of the American College of Cardiology Foundation, the American Heart Association, the European Cardiac Arrhythmia Society, the European Heart Rhythm Association, the Society of Thoracic Surgeons, the Asia Pacific Heart Rhythm Society, and the Heart Rhythm Society. Heart Rhythm.

